# Reactive vaccination in the presence of disease hotspots

**DOI:** 10.1098/rspb.2014.1341

**Published:** 2015-01-07

**Authors:** Andrew S. Azman, Justin Lessler

**Affiliations:** Department of Epidemiology, Johns Hopkins University Bloomberg School of Public Health, 615 North Wolfe St., Baltimore, MD 21205, USA

**Keywords:** infectious disease transmission, epidemiology, communicable disease control, cholera, reactive vaccination, outbreak response

## Abstract

Reactive vaccination has recently been adopted as an outbreak response tool for cholera and other infectious diseases. Owing to the global shortage of oral cholera vaccine, health officials must quickly decide who and where to distribute limited vaccine. Targeted vaccination in transmission hotspots (i.e. areas with high transmission efficiency) may be a potential approach to efficiently allocate vaccine, however its effectiveness will likely be context-dependent. We compared strategies for allocating vaccine across multiple areas with heterogeneous transmission efficiency. We constructed metapopulation models of a cholera-like disease and compared simulated epidemics where: vaccine is targeted at areas of high or low transmission efficiency, where vaccine is distributed across the population, and where no vaccine is used. We find that connectivity between populations, transmission efficiency, vaccination timing and the amount of vaccine available all shape the performance of different allocation strategies. In highly connected settings (e.g. cities) when vaccinating early in the epidemic, targeting limited vaccine at transmission hotspots is often optimal. Once vaccination is delayed, targeting the hotspot is rarely optimal, and strategies that either spread vaccine between areas or those targeted at non-hotspots will avert more cases. Although hotspots may be an intuitive outbreak control target, we show that, in many situations, the hotspot-epidemic proceeds so fast that hotspot-targeted reactive vaccination will prevent relatively few cases, and vaccination shared across areas where transmission can be sustained is often best.

## 1. Introduction

Reactive vaccination has become an important tool in the fight against diseases such as measles [1], meningitis [2], foot-and-mouth disease [3] and cholera [4]. Reactive vaccination campaigns are in a race with the natural epidemic timeline, and will avert few cases if started late or inappropriately focused [1,5]. In contrast to regularly planned vaccination campaigns, reactive campaigns are often subjected to limited vaccine, and logistical delays forcing vaccinators to make difficult allocation decisions.

The two internationally licensed oral cholera vaccines (OCVs) have an efficacy against clinical disease of over 65% lasting at least 3–5 years [6,7]. In 2012, 245 393 cholera cases were reported to the World Health Organization (WHO) including 3034 deaths [8]. Owing to non-reporting and under-reporting in many high burden countries, this number is known to be a serious underestimate [9]. To ensure that a minimal supply of OCV is available for rapid deployment, a stockpile large enough to vaccinate one million individuals was established by the WHO. However, with an estimated 1.4 billion people at risk for cholera [9], the current supply is insufficient to reach even a small fraction of these people in a given year. The WHO has developed criteria for vaccine request prioritization [10]; however, many questions remain on how to make vaccine allocation decisions at the local and global scale.

The targeting of ‘hotspots’ has been suggested as one potential mechanism for efficiently using limited vaccine. ‘Hotspots’ are areas of increased transmission potential or incidence and have been described for cholera and other diseases at spatial scales ranging from countries within a region [11], to districts within a country [12], to neighbourhoods within a city [5,13]. Here, we ask if, and when, the targeting of transmission hotspots represents the best use of limited vaccine.

The performance of different vaccine allocation strategies is driven by a number of complex factors, including heterogeneity in transmission potential across the area, the immune landscape [14,15] and the epidemiologic connectivity (i.e. spatial coupling) between infected areas. For many diseases, epidemiologic connectivity is driven by human movement; though transmission through vectors or the environment may also play an important role. Connectivity between areas may be measured empirically (through analysis of cell phone data, etc. [16]), but is usually approximated using models of human movement [17–19]. In general, all approaches demonstrate high connectivity between areas close to one another (e.g. neighbourhoods within cities), and low connectivity between spatially disparate areas, particularly if both have small populations.

While previous publications have estimated the optimal proactive allocation of vaccine in specific settings [15,20,21], it may be unrealistic to presume that such optimality analyses would be performed as part of a real-world public health response, as they require complex analyses based on highly detailed information about transmission dynamics. Here, we build upon the results of previous work, which mostly focuses on influenza vaccination strategies [15,20–22], to show how three intuitive and simple reactive vaccination strategies may perform over different epidemiologic landscapes when vaccine supply is limited. We translate our results in a manner that can help build intuition for when different strategies may work best; particularly when the availability of data and resources constrain the level of analyses that can be performed.

**Figure 1.**
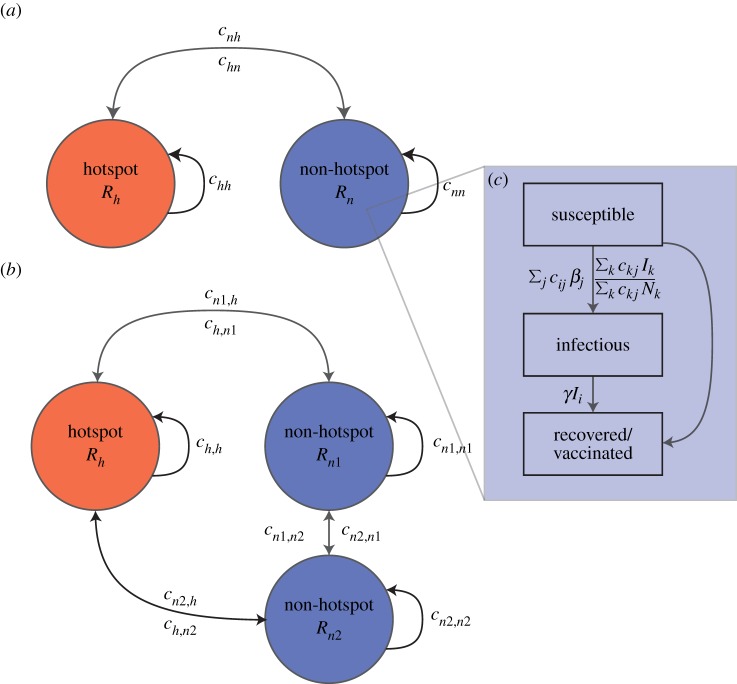
Metapopulation configuration for simulations with 1 (*a*) and 2 (*b*) non-hotspots and a single hotspot. Each patch is represented by a circle with population proportional to area (equal population sizes shown). The local basic reproductive number for each area is represented by *R_i_*, and the edges represent the parameters of the connectivity matrix **C**. Panel (*c*) illustrates the main transmission model within each patch. Boxes represent states, and edges show the rates of transition from one state to another. (Online version in colour.)

Here, we use simple metapopulation models of a cholera-like disease (figure 1) to explore how to allocate vaccine both proactively and reactively when supply is limited in the presence of a transmission hotspot and one or more non-hotspots. We estimate the reduction in epidemic size achieved by three simple strategies over a range of epidemiologic settings: (i) targeting vaccine at the transmission hotspot, (ii) targeting vaccine at non-hotspot(s) and (iii) allocation proportional to population size (pro-rata).

## 2. Methods

### (a) Transmission model

We use deterministic SIR metapopulation models to estimate the trajectory and final epidemic size with and without vaccination. The basic model can be described by a system of ordinary differential equations as follows2.1
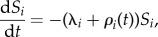
2.2
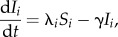
2.3
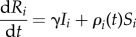
2.4
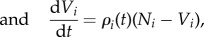
where the states, susceptible, infectious and removed are represented by *S*, *I* and *R*, respectively with the subscript, *i*, indicating the patch that the individuals reside within. For example, *S_i_* represents the number of susceptible individuals in patch *i*. We also use a redundant-state variable, *V*, to track the number of individuals vaccinated within this model, regardless of their immune status when vaccinated. The force of infection, *λ_i_*, driven by the transmission parameters (*β_i_*) for each patch, is formulated to maintain frequency-dependent transmission (i.e. contact rates are independent of population size) as2.5
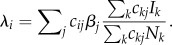
Mixing between patches is described by the connectivity matrix **C**, where each term (*c_ij_*) represents the average proportion of time an individual from patch *i* spends in patch *j. N_i_* represents the number of individuals (*S_i_* + *I_i_* + *R_i_*) in location *i*. The recovery rate is represented by *γ* and is assumed to be 1/3 days^−1^ [23].

The patch-specific vaccination rate, *ρ_i_*(*t*), is defined such that vaccination takes place over a fixed number of days and vaccinated individuals are not at risk for revaccination2.6
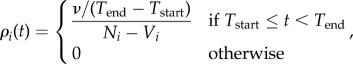
where *T*_start_ and *T*_end_ are the start and end day, respectively, of the vaccination campaign, and *ν* is the total number of vaccine courses available. We assume that the full vaccine course instantaneously confers complete protection to all vaccinated (VE*_sp_* = 1 [24]), and that vaccination campaigns are completed in a single day. More complex vaccine and vaccination campaign characteristics can easily be captured by this model, but we restrict ourselves to this simple case to help elucidate the main results. Imperfect vaccines are explored in the electronic supplementary material, figures S13 and S14.

In this model, the basic reproductive number, 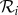, for an unconnected patch is *β_i_*/*γ*, and the basic reproductive number for the total connected system can be found using the dominant eigenvalues of the next-generation matrix (see the electronic supplementary material) [25].

We first consider settings with two 500 000 person populations (patches) with varying levels of symmetric connectivity and internal transmission potential (i.e. 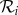). We explore settings where patches range in their connectivity, from unconnected (*c_ij_* = 0.0 for all *i* ≠ *j* and *c_ii_* = 1.0), where there is no movement between patches, to highly connected (*c_ij_* = 0.2 for all *i* ≠ *j* and *c_ii_* = 0.8) where 20% of the population in one patch are, on average, in other patches at any one time (electronic supplementary material, table S3 and figure 1). We consider metapopulations where the reproductive numbers for an individual patch range from 0.75 to 2.5—encompassing the majority of estimates of the reproductive number for cholera in different settings [5,12,26,27] and explore higher reproductive numbers in sensitivity analyses. In each scenario, we allow one patch to have a higher 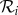 than the others and call this the ‘hotspot’ and the others ‘non-hotspot(s)’, and simulations are seeded with a single case in each patch. In the primary simulations, the populations are considered to be fully susceptible to the disease but we explore the impact of more realistic immune landscapes through simulating 40 years of epidemics recurring every 4 years where individuals who gain immunity through infection or vaccination lose their immunity after an average of 4.5 years; an approximation consistent with the estimated duration of protection from oral cholera vaccine (5 years, [6]) and an average lifespan of 50 years.

### (b) Proactive vaccination

We use a simple probabilistic representation of the final size of a generic epidemic in a metapopulation (following Longini *et al*. [28]) to derive analytical results for the impact of different proactive vaccination strategies (see the electronic supplementary material). The final size predicted by these models has been shown to be robust to many assumptions of simple SIR models [29].

### (c) Reactive vaccination

We explore the final size of epidemics as the amount of vaccine available increases from 0 to 500 000 courses (enough for one entire patch) with different simple allocation strategies; (i) targeting the hotspot, (ii.a) targeting a single non-hotspot, (ii.b) targeting all non-hotspots and (iii) pro-rata vaccination. For each scenario (electronic supplementary material table S3), we compare the strategies by calculating the percentage difference between the strategies that averts the fewest and most cases, *Θ*2.7
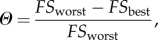
where *FS*_best_ and *FS*_worst_ are the epidemic final sizes using the strategies that averted the most (best) and fewest (worst) cases. This statistic is useful for comparing strategies, but gives no information on the number of cases averted in any of the simulations. All simulations were performed in Python, and visualizations were created with R [30,31]. Source code will be made available by the authors upon request.

## 3. Results

### (a) Proactive vaccination

**Figure 2.**
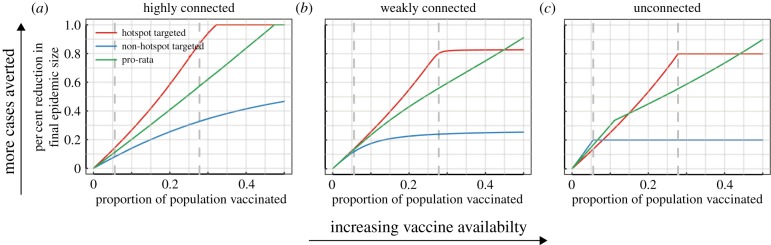
(*a*–*c*) Comparison of three proactive vaccination strategies in populations with decreasing levels of connectivity (across columns *α* = 0.2, 0.01, 0.0, respectively from left to right). In these simulations, 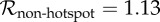 and 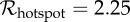. The dashed lines represent the amount of vaccine necessary to achieve the critical vaccination threshold through targeting each population (left most is for the non-hotspot on each plot).

When vaccinating proactively in highly connected settings, such as neighbourhoods within a city, targeting the hotspot is preferable, regardless of how much vaccine is available (figure 2*a* and the electronic supplementary material, S3). Vaccination in unconnected settings should be targeted at the non-hotspot when doses are limited (i.e. until there are at least enough doses to bring the reproductive number below one in the non-hotspot, but not enough to do so in the hotspot), but the best strategy then switches to pro-rata followed by hotspot-targeted vaccination as more vaccine becomes available (figure 2*c*). At the highest vaccination levels considered, the best strategy transitions back to pro-rata. The best strategies in weakly connected settings look similar to those of unconnected settings although the relative difference between strategies is smaller (figure 2*b*).

### (b) Reactive vaccination

#### (i) Timing

**Figure 3.**
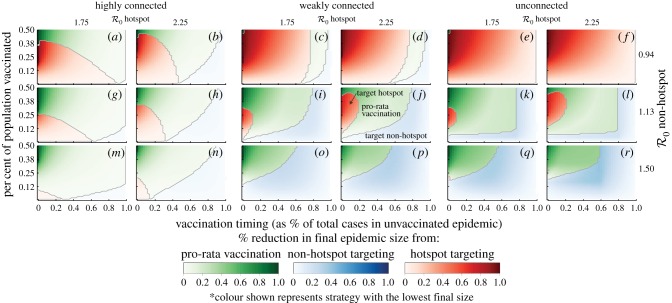
(*a*–*r*) Overview of relative performance of vaccination strategies in two-patch system. Panels illustrate best vaccination strategies (as measured by *Θ*) as a function of (i) availability of vaccine (*y*-axis, as a per cent of the total population), (ii) the timing of the vaccination campaign (*x*-axis, as the per cent of total cases infected in an uncontrolled epidemic) and (iii) the transmission efficiency (as measured by the (local) reproductive number, 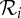) in each patch. The colour of each grid cell represents the preferred strategy at that vaccine availability level and vaccination campaign timing (green for pro-rata, blue for non-hotspot targeting, red for hotspot targeting), with the colour intensity representing, *Θ*, or how much better that strategy is than the worst strategy (darker colours representing situations where the best decision is far better than the worst). Solid grey lines represent shifts in the optimal strategy. Colours are not overlaid on one another, and only the colour of the best strategy is displayed in any one pixel. Alternative displays of these data are available in the electronic supplementary material.

**Figure 4.**
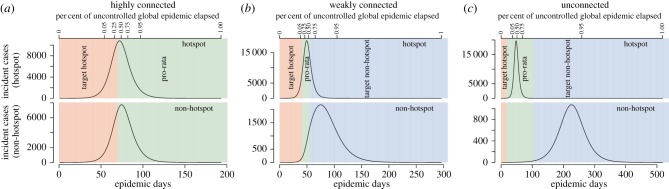
Epidemic curves for hotspot (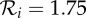) and non-hotspot (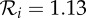) and timings for switching to different vaccination strategies. The populations in these simulations were symmetrically connected with *c_ij_*(*i* ≠ *j*) = 0.2, 0.01, 0.0 (and *c_ii_* = 1 − *c_ij_*), from left to right (*a–c*), respectively, with 13.8% of the total population vaccinated (same simulations as panels *g*, *i* and *k* in figure 3). The *y*-axes are of different scale to highlight the shape of the different epidemic curves. Shaded areas represent the zones where a specific strategy is preferable with red representing hotspot-targeted vaccination, green representing pro-rata vaccination and blue representing non-hotspot-targeted vaccination. It should be noted that this scenario illustrates a small part of the decision space where all three strategies are optimal at some time throughout the epidemic.

We find that, when vaccine is limited, the best strategies for proactive vaccination differ significantly from those for reactive vaccination (i.e. vaccination after the start of the epidemic; figure 3 and the electronic supplementary material, S7 and S11). A general pattern emerges from our simulations where, over the course of the epidemic, hotspot-targeted vaccination is first preferred, then pro-rata vaccination, and finally targeting the non-hotspot (figure 3 and the electronic supplementary material, S7). However, depending on the connectivity and transmission potential, the continuum of optimal strategies may begin where pro-rata or non-hotspot targeting is preferred (e.g. figure 3*o*), or never transition from a point where hotspot targeting is preferred (e.g. figure 3*f*). In our simulations, the point at which hotspot targeting was replaced by pro-rata vaccination was before the global epidemic peak (e.g. figure 4) except for cases where the non-hotspot had an 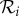 close to that of the hotspot. Thus, if the global peak has occurred, more cases will be averted if some vaccine is distributed to the non-hotspots, provided they can independently sustain transmission (i.e. 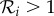).

As more non-hotspot patches are considered, hotspot-targeted vaccination is preferable until a smaller proportion of the uncontrolled epidemic has elapsed. However, because the addition of multiple non-hotspots makes the global epidemic slower (electronic supplementary material, table S1), the time window for hotspot vaccination is actually longer on a natural timescale (e.g. days, electronic supplementary material, figure S10).

In all situations, we find that the later the vaccination begins, the smaller the relative difference between strategies. In highly connected settings, the relative performance of the best strategies quickly dissipates as vaccination starts later, despite the fact that many cases may be averted (figure 3 and the electronic supplementary material, S8 and S11).

#### (ii) Connectivity

The degree to which patches are connected also plays a large role in shaping the impact of reactive vaccination strategies. Increasing connectivity serves two functions, (i) it reduces the indirect benefits from others vaccinated in a person's patch because of the increased risk of infection from outside the patch, and (ii) it helps to synchronize the timescales of the epidemic in different areas. The relative benefit of the best vaccination strategies shrinks as populations become more connected (figure 3 and the electronic supplementary material, figure S7). While targeting may be most operationally relevant in highly connected populations (e.g. within a city), the importance of connectivity on dynamics should still be understood.

When patches can sustain transmission, decreasing connectivity shifts the optimal vaccination strategies from hotspot-targeted and pro-rata (e.g. figure 3*g*, *l* and *k*) to non-hotspot-targeted and pro-rata (figure 3*g*–*r* and electronic supplementary material, figure S7*g*–*j*). Decreasing connectivity leads to larger impacts from allocating vaccine to the non-hotspot. In unconnected settings, when not enough vaccine is available to meet the critical vaccine threshold in the non-hotspots [32], non-hotspot-targeted vaccination is preferred, regardless of timing (figure 3 and electronic supplementary material, figure S8). A similar pattern occurs in weakly connected settings, though the transition from preferring non-hotspot-targeted to pro-rata vaccination happens at fewer doses. Pro-rata vaccination is preferred early on in the epidemic for unconnected and weakly connected setting, with hotspot targeting only preferred in a few cases where vaccination is essentially proactive or when non-hotspots cannot sustain transmission (figures 3–4 and the electronic supplementary material, figure S8). If vaccine must be targeted to a single patch for operational reasons, similar connectivity and timing trade-offs occur (electronic supplementary material, figure S7).

#### (iii) Transmission potential heterogeneity

The local basic reproductive numbers play important roles in shaping the outcomes of different vaccination strategies. When transmission cannot be supported in non-hotspots, vaccination should usually be targeted at the hotspot (figure 3*a*–*f*). When the reproductive number of the non-hotspots is close to the threshold value of 1 (e.g. figure 3 and electronic supplementary material, S8*g*–*l*), which may often be the case with cholera [5], early vaccination aimed at the hotspot or pro-rata is usually preferable. As the non-hotspot reproductive number approaches that of the hotspot, the preferred strategy shifts away from hotspot targeting towards pro-rata and non-hotspot targeting (figure 3 and electronic supplementary material, figure S8). In settings where the non-hotspot reproductive number is well above 1, hotspot-targeted vaccination is almost never preferable, and when it is, the relative difference between hotspot targeting and any of the other strategies is very small (e.g. 11%, 7%, 5% for 3, 4 and 5 patch models in the electronic supplementary material, figure S9 where 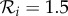 in the non-hotspot).

## 4. Discussion

Vaccination plays an increasing role as an outbreak response tool throughout the world [2–4]. Its success hinges on our ability to minimize surveillance and logistic delays while targeting appropriate subpopulations [1,5]. Through simple computational models, we have shown that while targeting transmission hotspots may be preferable in many proactive vaccination campaigns, reactive hotspot targeting may avert fewer cases than other simple allocation strategies. Epidemiological context matters, and in particular, the connectivity between populations, their transmission efficiency and the amount of vaccine available all shape the outcomes of reactive vaccination strategies.

In highly connected areas, akin to neighbourhoods in a city, early allocation of at least some vaccine to the transmission hotspot will help avert relatively more cases than other strategies (figure 4*a*). As vaccination is delayed, allocation focus should shift towards non-hotspot areas, where the population is more likely to still be susceptible to the disease. In less connected settings, targeted vaccination at the transmission hotspot is unlikely to avert the most cases. Instead, priority should be given to strategies that share vaccine between areas (e.g. pro-rata) if vaccination starts early, with the preference then shifting towards targeting one or more non-hotspot populations (figure 4*b*,*c*).

The simplicity of our simulations limits the generalizability of the results. We present results for metapopulations comprised of two to five patches; however, in practice, many more populations will be contenders for vaccination and multiple areas may serve as hotspots. However, populations with similar transmission potential may be grouped together creating scenarios similar those in this manuscript. When non-hotspots have different 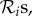
those that are able to independently sustain transmission should be targeted. Populations that may be vaccinated will rarely be the same size with the same immune landscape, nor will epidemics start at the exact same time. However, this simplification is useful in understanding the trade-offs involved with vaccine allocation decisions. In a sensitivity analysis, we explored the impact of vaccination on partially immune landscapes and our qualitative findings remain intact (electronic supplementary material, figure S6). In this model, we consider only the impact of vaccination on a single epidemic. In reality, many vaccines, including OCV [6], may last long enough to confer immunity to diseases that periodically reoccur. If vaccinating within an area with periodic outbreaks, some weight should be given to the potential effect of vaccine-derived protection on future epidemics.

For the sake of clarity, we use a simple model of vaccination that includes a perfect vaccine that instantly provides protection. Oral cholera vaccines are given as a two-dose regimen, and maximum protection is thought to occur days after the second dose [33]. Our results are presented in a manner that can allow for approximation of the best strategy in the presence of an imperfect vaccine by interpreting the *y*-axis (of figure 3) as the proportion protected rather than the proportion vaccinated (i.e. looking at coverage × vaccine efficacy); however, this approximation deviates slightly from the exact results (electronic supplementary material, figures S13 and S14). Similarly, the effect of delays in the onset of protection can be seen by considering the timing of vaccine-derived protection rather than timing of the vaccination campaign (e.g. the *x*-axis in figure 3).

To allow for comparisons between epidemics on different timescales, we present the timing of vaccination campaigns in terms of the percentage of the uncontrolled epidemic elapsed. While this time metric is useful across different settings, it is more difficult to interpret in the context of a specific epidemic. With the basic epidemics simulated, one can roughly map key percentiles to different features of the local epidemic curves. In highly connected settings, usually the first 25% of uncontrolled epidemic will occur before the peak in both the hotspot and non-hotspot (see electronic supplementary material, figure S2). The point at which half of the uncontrolled cases have been infected tends to occur just after peak, with this point coming relatively earlier in the non-hotspot(s) than the hotspot. As epidemics become less connected, this mapping between natural time and percentage of uncontrolled epidemic elapsed becomes more complex and heavily depends on the number of patches considered. In all settings simulated, the first 10% of cases always occur before the peak in both the hotspot and the non-hotspot, and usually occur during the initial period of ‘exponential’ growth. The decision of when to target the hotspot, in situations where all areas are able to sustain transmission, will often come down to identifying whether one has a reasonable amount of vaccine, and whether vaccination can begin very early in the epidemic (often less than 10–20% of the uncontrolled epidemic size elapsed). In these situations, this basic mapping between percentiles and the peak of the epidemic should be sufficient to allow for application of our results.

This work compliments other research in the area of optimal vaccine use, including papers describing similar optimal allocations for proactive [14,15,20,34] and reactive [21,35] vaccination with some under moderate supply constraints. Our work extends these by focusing on reactive vaccination with realistic delays when transmission is spatially heterogeneous and only low vaccine coverage is attainable. Here, our goal is not to provide an algorithm for optimal allocation of specific numbers of vaccine doses, but instead to identify some of the key factors that should be considered when making strategic vaccine allocation decisions. While the relative connectivity between potential vaccine locations may be roughly known, the reproductive number in each population cannot be precisely known at the time of the epidemic. Use of historical data and real-time techniques for estimating 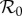 represent an exciting area of new research, and recently proposed methods may provide sufficient precision for identifying hotspots and deciding between simple allocation strategies [36]. Our results can also serve as a benchmark for the level of precision required for new methods to be useful for vaccine allocation decisions.

While we consider spatial-targeting strategies, others have used similar theory to explore optimal strategies for targeting by age or demographics who exhibit differences in infectiousness and susceptibility to severe disease [14]. In this context, targeting the groups most responsible for transmission is generally only preferred when vaccine is allocated very early on in an epidemic when transmission potential is high. Otherwise, mixed strategies that focus at least some vaccine towards those at highest risk for severe outcomes is optimal. Extensions of our work could incorporate both individual level heterogeneity in infectiousness (i.e. super spreaders) and susceptibility with location-specific transmission heterogeneity (e.g. in the case of cholera, this may be created through differential water and sanitation infrastructure) to better understand the trade-offs between spatial-targeting, demographic-targeting, combined strategies.

Additional research is needed to translate the results presented here into guidance that can be used in the midst of an outbreak. Our results do however suggest a general rule of thumb: in highly connected settings, reactive vaccination distributed across areas that can independently maintain transmission is generally preferred to targeting any particular transmission hotspot, and hotspots should be targeted only when they are thought to be necessary drivers of transmission. Although decision-makers are unlikely to be certain about whether areas can independently sustain transmission, combining real-time incidence and historical data with an understanding of the pathogen biology may allow for a reasonable estimate.

Like many health policy decisions, vaccination allocation strategies are rarely made based on epidemiologic predictions alone, but are also driven by social and political factors. Dedicating all available vaccine to a particular location and depriving other at-risk populations may be politically unpopular or raise equity issues, thus, these findings should be used as a general guide to be weighted along with other factors. Although we focused on situations with limited vaccine, our findings can also help guide vaccination strategies guided by cost-effectiveness thresholds.

Our results illustrate that the preferred vaccine allocation strategy, in the presence of limited vaccine supply and a transmission heterogeneity, varies depending on epidemiologic context and logistical constraints. Strategies that may be optimal before the start of an epidemic will usually not be optimal in reactive vaccination campaigns. In most cases, starting vaccination early is much more important than choosing between allocation strategies, particularly when choosing between areas that are expected to have the ability to sustain disease transmission. While targeting disease hotspots may sound like an intuitive strategy, in many cases, it makes more sense to focus on areas of lower transmission efficiency. These findings highlight the benefits and risks of targeting vaccination efforts when only a limited number of people can be vaccinated and should be considered carefully as part of the planning process.

## Supplementary Material

Supplementary Materials
