# Supplementary material for: Reactive vaccination in the presence of disease hotspots
Source: Proc Biol Sci. 2015 Jan 7;282(1798):20141341. doi: 10.1098/rspb.2014.1341 (PMC4262159; doi:10.1098/rspb.2014.1341)
Supplement: Supplementary Materials [file rspb20141341supp1.pdf]

Reactive Vaccination in the Presence of Disease Hotspots

Electronic Supplementary Material

Andrew S. Azman and Justin Lessler

Contents

1 Uncontrolled Epidemics Overview Tables and Figures **2**

2 Estimation of Metapopulation Reproductive Number **5**

3 Final Size for Proactive Vaccination **6**

4 Two Patch System **8**

5 Multiple Non-hotspots **12**

6 Final Size Reduction from Best Strategy **15**

7 Optimal Allocations in Two Patch Models **16**

8 Results with Imperfect Vaccines **17**

1 Uncontrolled Epidemics Overview Tables and Figures

Uncontrolled epidemic dynamics are a function of connectivity and patch-specific transmission efficiency ($\mathcal{R}_i$, the 'local' basic reproductive number). Simulated uncontrolled epidemics lasted from 12 to 86 weeks, with highly connected epidemics lasting longer as the number of non-hotspot patches increased (Table S1). The final size of these epidemics ranged from 163,704 to 1,677,912, with more heterogeneity in larger systems (i.e. those with more patches, Table S2). As a proportion of the total population size, uncontrolled epidemics infected 7-78% of the total population. Local epidemics (i.e. within a single patch) lasted as short as 20 days. Local final epidemic sizes ranged from 3 to 446,322 persons infected. The time to hotspot peak incidence, the first local epidemic peak that will occur in all scenarios, ranged from 25 to 240 days.

Table S1: Minimum and maximum durations (D) for global and local uncontrolled epidemics.

# Patches	Population	$D_{global,min}$	$D_{global,max}$	$D_{local,min}$
2	1,000,000	81	519	20
3	1,500,000	81	537	20
4	2,000,000	81	568	20
5	2,500,000	81	602	20

Table S2: Minimum and maximum final sizes (FS) for global and local uncontrolled epidemics.

# Patches	Population	$FS_{global,min}$	$FS_{global,max}$	$FS_{local,min}$	$FS_{local,max}$
2	1,000,000	243,609	783,559	3	446,322
3	1,500,000	199,927	1,086,449	3	446,322
4	2,000,000	177,124	1,383,293	3	446,322
5	2,500,000	163,704	1,677,912	3	446,322

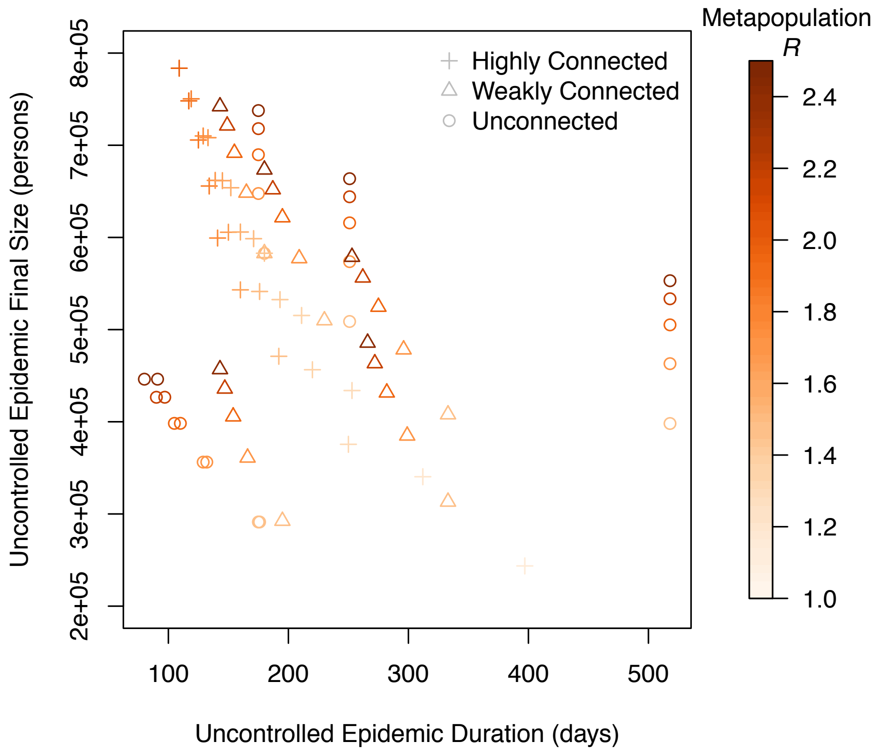

Figure S1: Final size and duration of uncontrolled epidemics in 2-patch system

Table S3: Overview of parameters used in simulations for main analyses

Parameter Description	Range
Epidemiologic Connectivity, c_{ij} $(i \neq j)$	$0.0 - 0.2$
Transmission potential hotspot, $\mathcal{R}^{hotspot}(unconnected)$	$1.5 - 2.5$
Transmission potential non-hotspot(s), $\mathcal{R}^{non-hotspot}(unconnected)$	$0.75 - 1.5$
Full vaccine courses	0-499,999
Vaccine timing (as percent of uncontrolled epidemic elapsed)	$0 - 1$

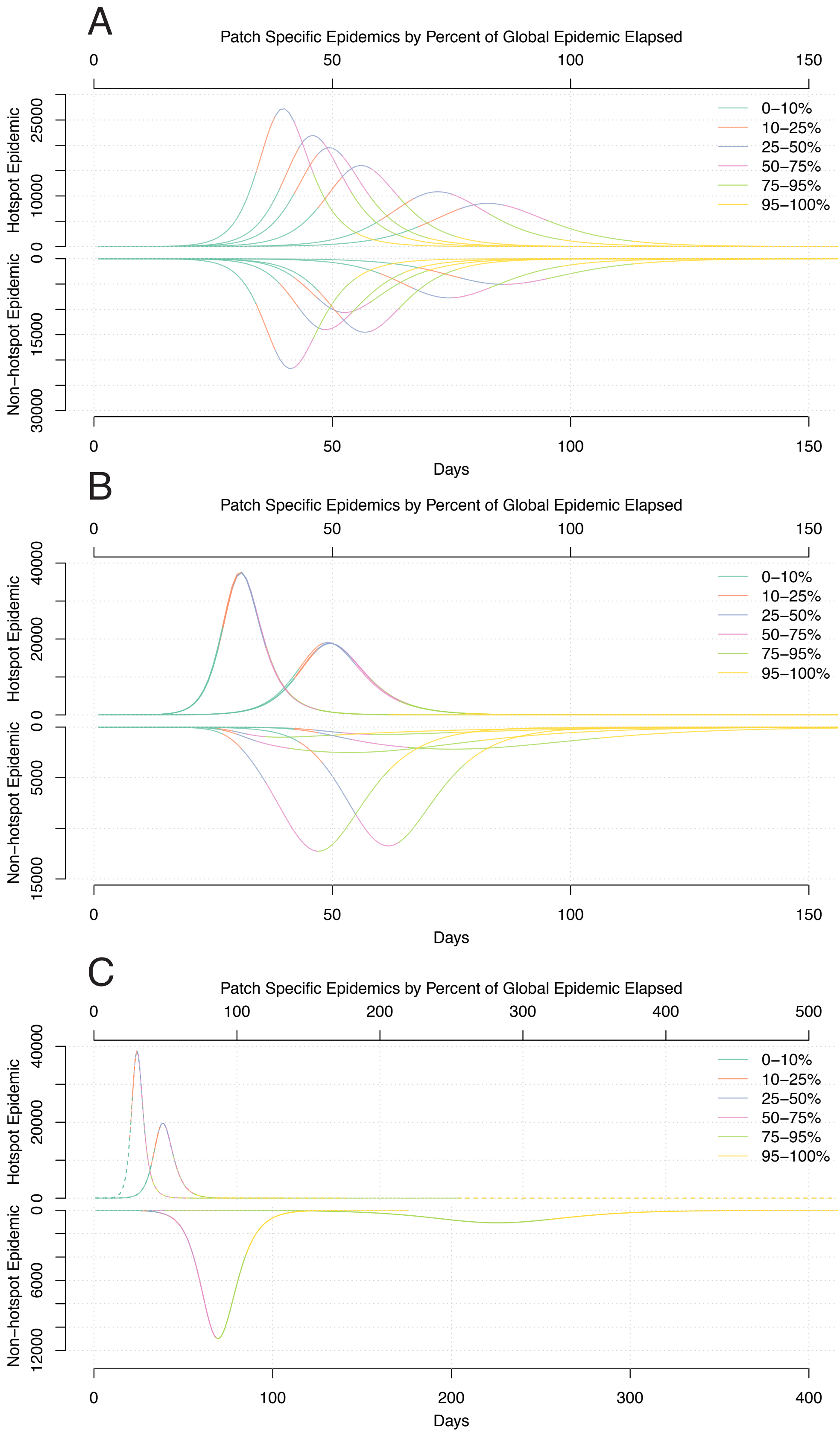

Figure S2: Hotspot (top) and non-hotspot (bottom) epidemic curves with sections coloured by percent of epidemic elapsed (two-patch model). Panels A, B and C show the curves for highly-connected, weakly connected, and unconnected settings shown in Figure 3 of the main text.

2 Estimation of Metapopulation Reproductive Number

In the main manuscript we use the reproductive numbers corresponding to unconnected populations (i.e. the reproductive number for a population had $c_{jj} = 1$) to help with comparisons. We can find the basic reproductive number for the whole metapopulation using the next generation matrix, [1] which is made up of two parts, the transmission matrix ($\mathbf{T}$), and transition matrix ($\mathbf{\Sigma}$). For our simple system with two populations the matrices are as follows:

$$\mathbf{T} = \begin{pmatrix} N_1\left(\frac{c_{11}^2\beta_1}{c_{11}N_1+c_{21}N_2} + \frac{c_{12}^2\beta_2}{c_{12}N_1+c_{22}N_2}\right) & N_1\left(\frac{c_{11}c_{21}\beta_1}{c_{11}N_1+c_{22}N_2} + \frac{c_{12}c_{22}\beta_2}{c_{12}N_1+c_{22}N_2}\right) \\ N_2\left(\frac{c_{11}c_{21}\beta_1}{c_{11}N_1+c_{21}N_2} + \frac{c_{12}c_{22}\beta_2}{c_{12}N_1+c_{22}N_2}\right) & N_2\left(\frac{c_{21}^2\beta_1}{c_{11}N_1+c_{21}N_2} + \frac{c_{22}^2\beta_2}{c_{12}N_1+c_{22}N_2}\right) \end{pmatrix} \quad (1)$$

$$\mathbf{\Sigma} = \begin{pmatrix} -\gamma & 0 \\ 0 & \gamma \end{pmatrix} \quad (2)$$

The next generation matrix, $\mathbf{K}$, is defined as $\mathbf{K} = -\mathbf{T}\mathbf{\Sigma^{-1}}$, and $\mathcal{R}$ is defined as the dominant eigenvalue of $\mathbf{K}$. Thus, in this simple system, we use the characteristic polynomial (and the quadratic formula) to get $\mathcal{R}$, as

$$\mathcal{R} = \frac{1}{2}\left(\text{tr}(\mathbf{K}) + \sqrt{\text{tr}^2(\mathbf{K}) - 4\det(\mathbf{K})}\right)$$

Table S4: $\mathcal{R}$'s for weakly connected populations $c_{ij} = c_{ji} = 0.01$ by unconnected reproductive number in the hotspot and non-hotpot

	Unconnected $\mathcal{R}_{non-hotspot}$				
Unconnected $\mathcal{R}_{hotspot}$	0.75	0.9375	1.125	1.3125	1.5
1.5	1.47	1.47	1.47	1.47	1.50
1.75	1.72	1.72	1.72	1.72	1.72
2	1.96	1.96	1.96	1.96	1.96
2.25	2.21	2.21	2.21	2.21	2.21
2.5	2.45	2.45	2.45	2.45	2.45

Table S5: $\mathcal{R}_i$'s for highly connected populations $c_{ij} = c_{ji} = 0.2$ by unconnected reproductive number in the hotspot and non-hotpot

Unconnected $\mathcal{R}_{hotspot}$	Unconnected $\mathcal{R}_{non-hotspot}$: 0.75	0.9375	1.125	1.3125	1.5
1.5	1.15	1.21	1.28	1.36	1.45
1.75	1.31	1.36	1.42	1.49	1.58
2	1.47	1.51	1.57	1.64	1.71
2.25	1.63	1.67	1.72	1.78	1.85
2.5	1.79	1.83	1.88	1.93	2.00

3 Final Size for Proactive Vaccination

We can use a simple probabilistic representation of the final size of an epidemic to explore the impact of proactive vaccination strategies with two populations. Here we derive the final size assuming the epidemic follows a Poisson process in a manner similar to previous studies [2, 3]. The final size in location i, Z^i_∞, can be represented by

$$Z^i_\infty = S^i_0 - S^i_\infty = S^i_0 \left(1 - \exp\left(-\sum_{j=1}^{n} \frac{\mathcal{R}^{i,j}_0 Z^j_\infty}{N_i},\right)\right) \tag{3}$$

where S^i_0 represents the initial susceptibles in population i, $\mathcal{R}^{i,j}$ represents the number of secondary infections in location i caused by an individual in location j , and N_i is the population in location i, and n is the number of locations (in this situation $n = 2$).

For simplicity, we explore models where the epidemic connectivity is defined by a single parameter α, that represents the proportion of subsequent infections an infectious individual makes outside his or her home area.

$$\mathcal{R}^{i,j}_0 = \begin{cases} \frac{\mathcal{R}^{j,j}_0 \alpha}{n-1} & \text{if } i \neq j \\ \mathcal{R}^{j,j}_0(1-\alpha) & \text{if } i = j \end{cases} \tag{4}$$

The final epidemic size after proactive vaccination is represented by an 'all or nothing' vaccine [4] that reduces the initial susceptible in the population as follows:

$$Z_{\infty}^{i}(v^{i}) = (S_{0}^{i} - \delta v^{i}) \left(1 - \exp\left(-\sum_{j=1}^{n} \frac{\mathcal{R}_{0}^{i,j} Z_{\infty}^{j}(v^{j})}{N_{i}},\right)\right) \quad (5)$$

where v^i represent the number vaccinated in area i and δ is the vaccine efficacy (VE_S). It should be noted that $Z_{\infty}^{i}(v^{i})$ is convex in v^i, a convenient property that can be exploited when trying to solve for the optimal allocation of vaccine.[5]

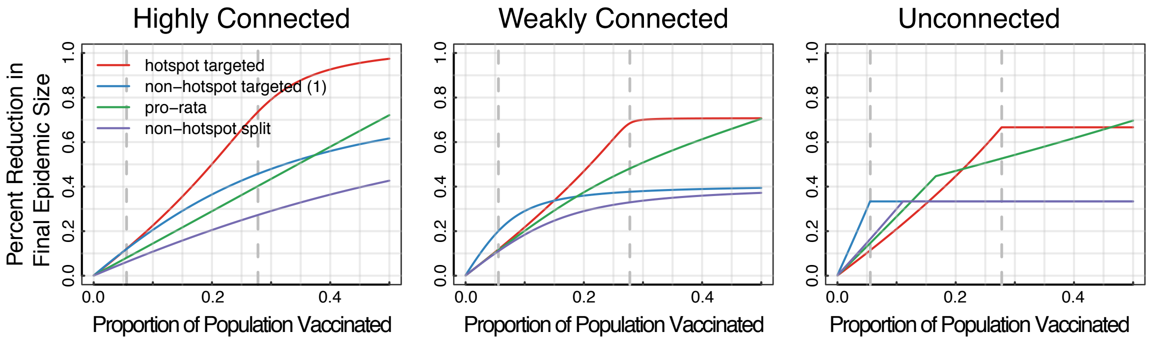

Figure S3: Comparison of three proactive vaccination strategies in a 3 patch model with with different levels of connectivity (across columns $\alpha = 0, 0.01, 0.2$, respectively from left to right). In these simulations $\mathcal{R}^{non-hotspot} = 1.13$ and each non-hotspot had a reproductive number of $\mathcal{R}^{hotspot} = 2.25$. The dashed lines represent the amount of vaccine necessary to achieve the critical vaccination threshold through targeting each population (left most is for the non-hotspot on each plot).

4 Two Patch System

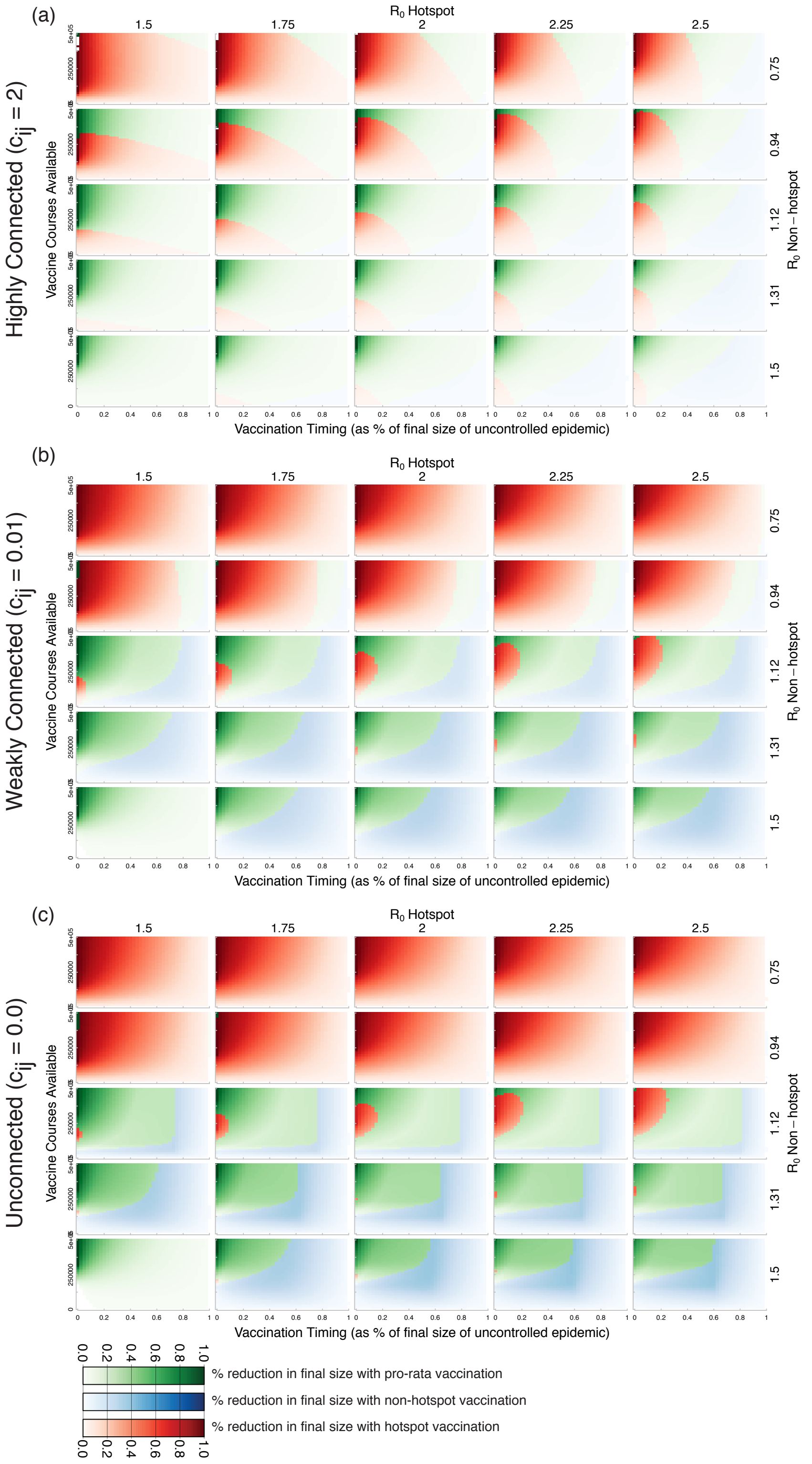

Figure S4: Comparison of simple reactive vaccination strategies in a 2-patch system with varying connectivity, $\mathcal{R}^{hotspot}$ and $\mathcal{R}^{non-hotspot}$. Colours represent the best strategy (red for hotspot-targeted, blue for non-hotspot-targeted, and green for pro-rata), and color intensity represent the percent difference between that and the worst strategy ($\Theta = \frac{FS_{worst} - FS_{best}}{FS_{worst}}$).

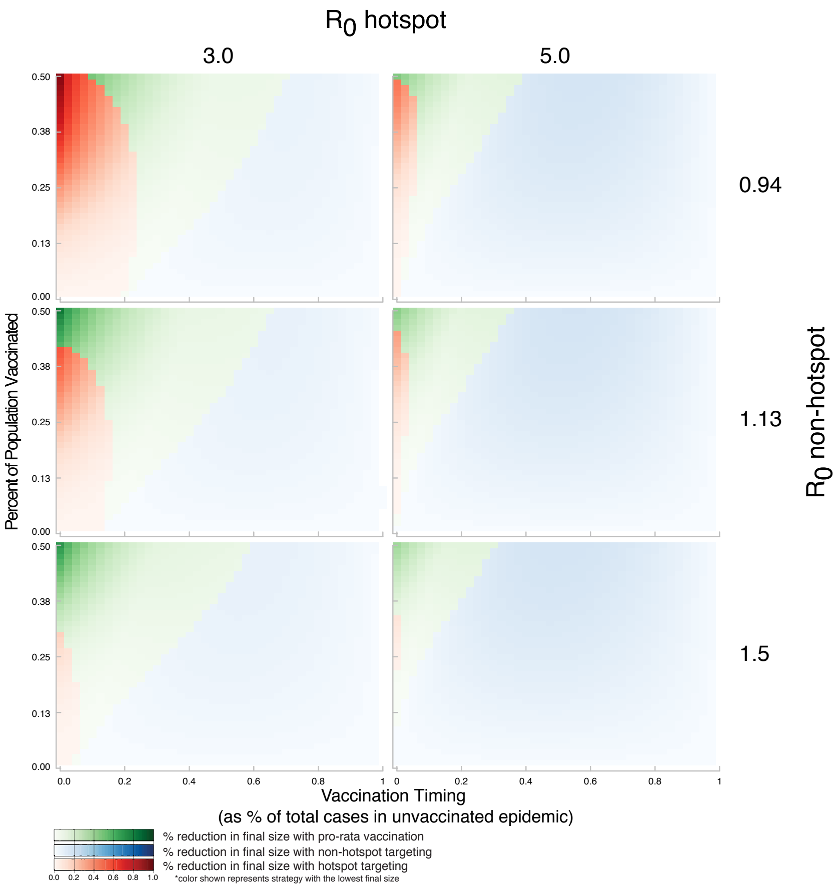

Figure S5: Comparison of simple reactive vaccination strategies in a 2-patch system with high reproductive numbers in the hotspot ($\mathcal{R}_{hotspot} = 3$ and 5) and high connectivity (0.2).

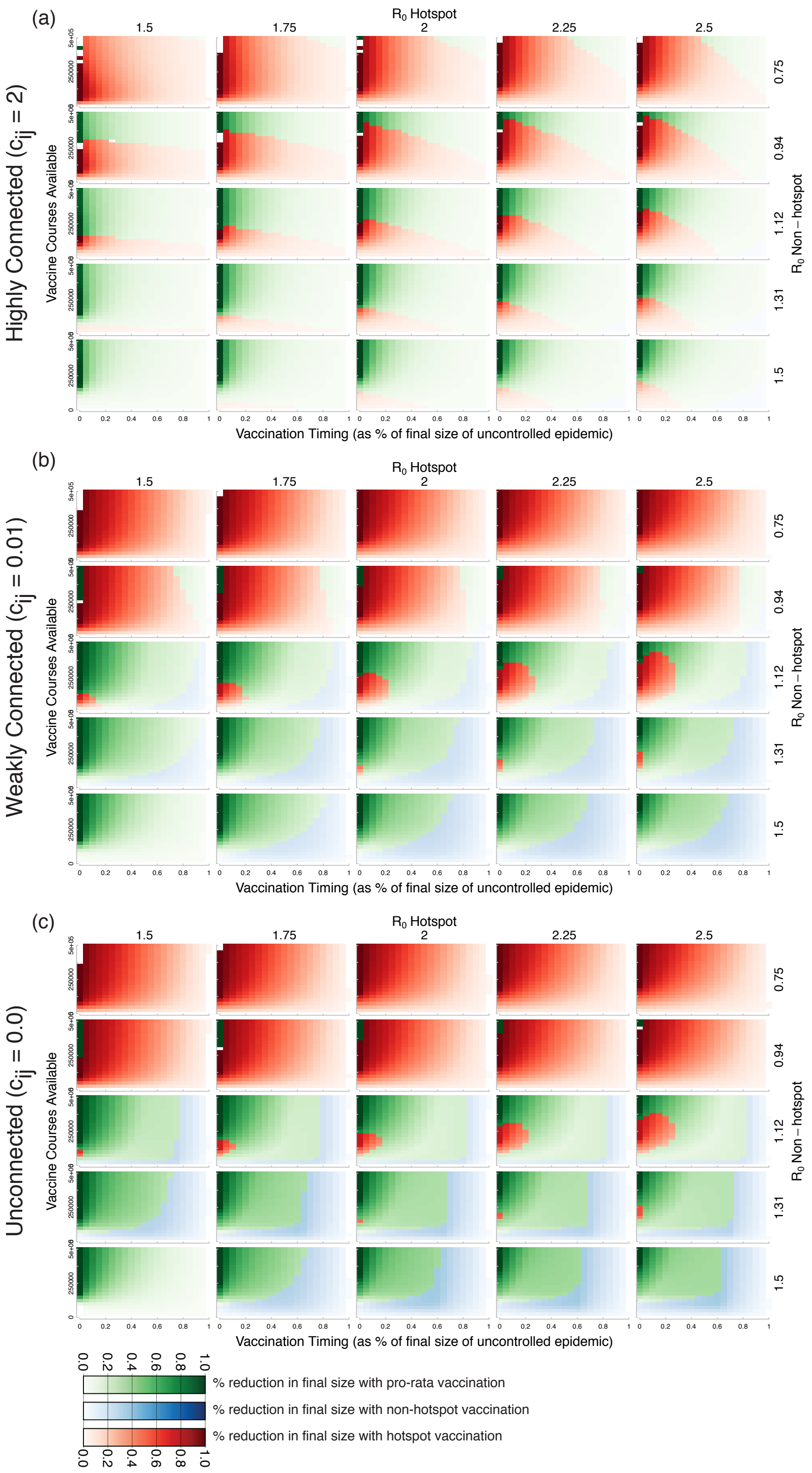

Figure S6: Comparison of simple reactive vaccination strategies in a 2-patch system with varying connectivity, $\mathcal{R}^{hotspot}$ and $\mathcal{R}^{non-hotspot}$ after 40 years of recurring epidemics every 4 years. These simulations assumed an exponential susceptible replacement distribution with a rate of $1/5+1/50$, thus every 4-years 58.5% of individuals who were immune from the previous epidemic become susceptible again. Colours represent the best strategy (red for hotspot-targeted, blue for non-hotspot-targeted, and green for pro-rata), and color intensity represent the percent difference between that and the worst strategy ($\Theta = \frac{FS_{worst}-FS_{best}}{FS_{worst}}$).

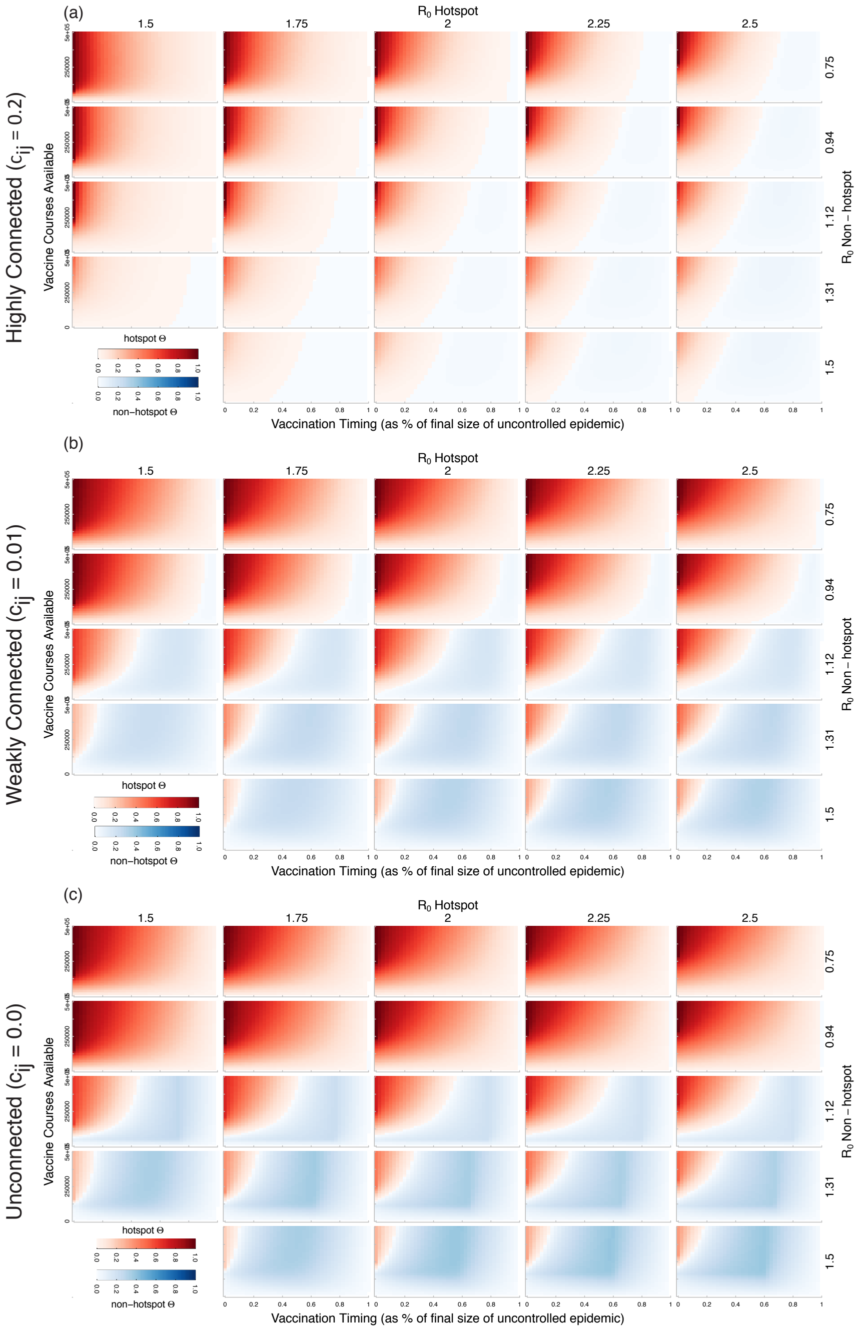

Figure S7: Comparison of vaccination targeted at the hotspot and vaccination targeted at the non-hotspot in a 2-patch system with varying connectivity, $\mathcal{R}_{hotspot}$ and $\mathcal{R}_{non-hotspot}$. Colours represent the best strategy (red for hotspot-targeted and blue for non-hotspot-targeted) and color intensity represent the percent difference between that and the worst strategy ($\Theta = \frac{FS_{worst}-FS_{best}}{FS_{worst}}$).

5 Multiple Non-hotspots

Now we consider metapopulations with 3-5 patches. In each set of simulations we allow there to be a single hotspot and multiple non-hotspot with identical R's. All sub-populations in these simulations are 500,000 individuals. We consider 4 different targeting approaches here; (1) targeting a single hotspot, (2) targeting a single non-hotspot, (3) sharing vaccine between all non-hotspots, and (4) pro-rata vaccination.

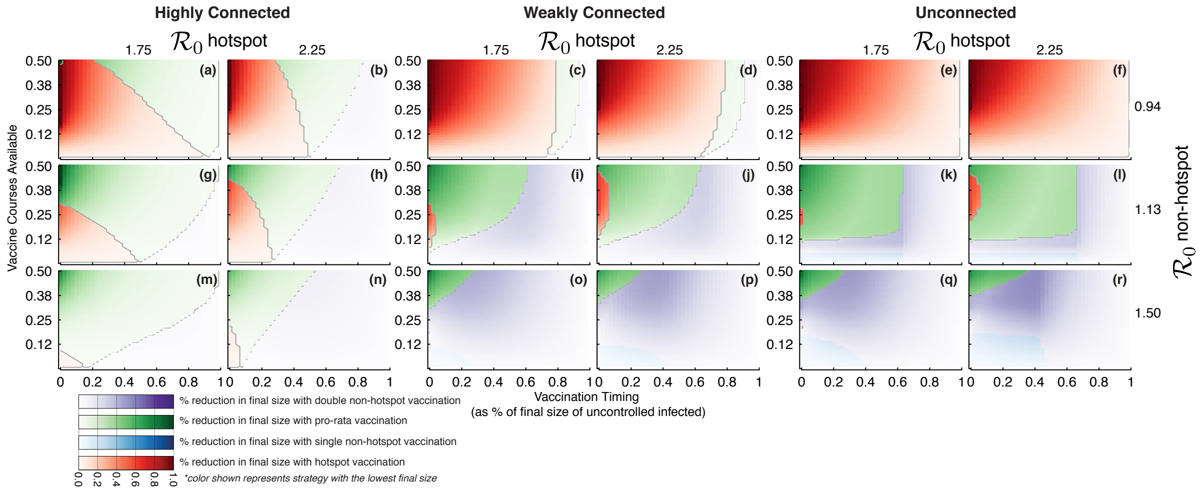

Figure S8: Overview of relative performance of vaccination strategies in 3-patch system. Panels illustrate best vaccination strategies (as measured by Θ) as a function of (1) availability of vaccine (y-axis, as a % of the total population), (2) the timing of the vaccination campaign (x-axis, as the percent of total cases infected in an uncontrolled epidemic), and (3) the transmission efficiency (as measured by the (local) reproductive number, $\mathcal{R}$) in each patch. The color of each grid cell represents the preferred strategy at that vaccine availability level and vaccination campaign timing (green $\rightarrow$ pro-rata, blue$\rightarrow$ single non-hotspot targeting, purple$\rightarrow$ shared non-hotspot targeting, red$\rightarrow$hotspot targeting), with the color intensity representing, Θ, or how much better that strategy is than the worst strategy (darker colors representing situations where the best decision is far better than the worst).

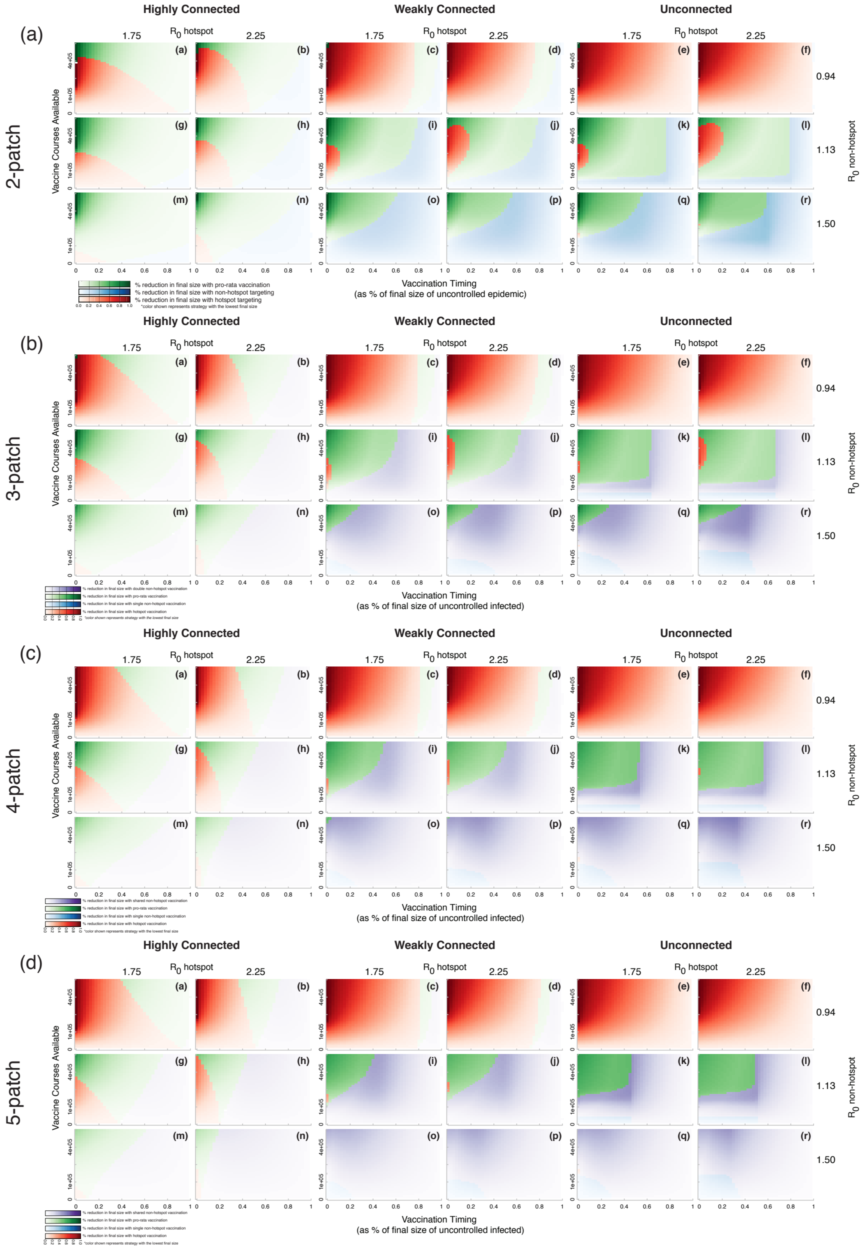

Figure S9: Relative performance of targeted and non-targeted vaccination strategies with a single hotspot and varying (2-4) number of non-hotspots. Colours represent the best strategy and color intensity represent the percent difference between that and the worst strategy ($\Theta = \frac{FS_{worst} - FS_{best}}{FS_{worst}}$).

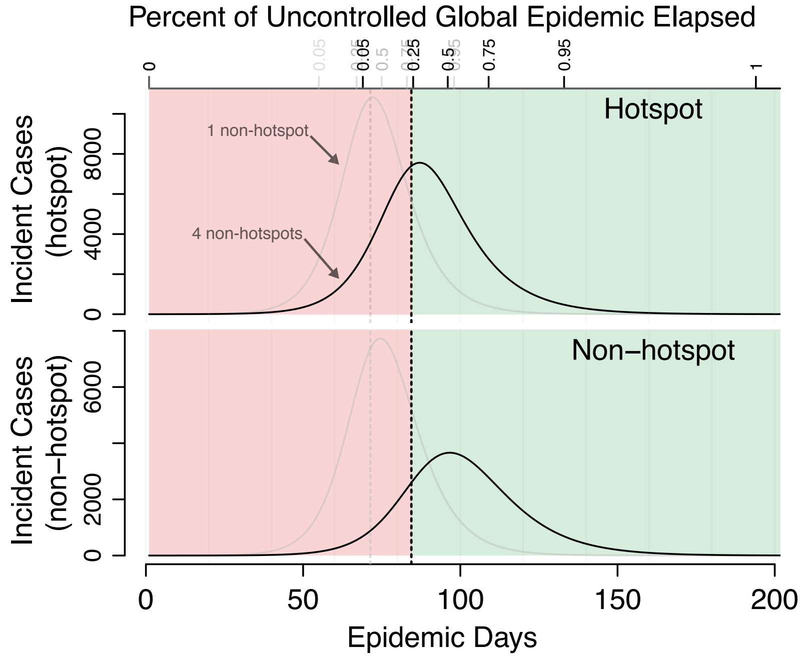

Figure S10: Illustration of the timings of optimal transition from hotspot targeting to pro-rata vaccination in highly connected 5-patch simulation with $\mathcal{R}_{hotspot} = 1.75$ and $\mathcal{R}_{non-hotspots} = 1.13$. The epidemic curves and decision threshold (dashed line) from a 2-patch model are shown in grey for comparison. Top x-axis shows the percent of uncontrolled epidemic elapsed (2-patch in grey, and 5-patch in black) and the bottom x-axis shows the number of days elapsed.

6 Final Size Reduction from Best Strategy

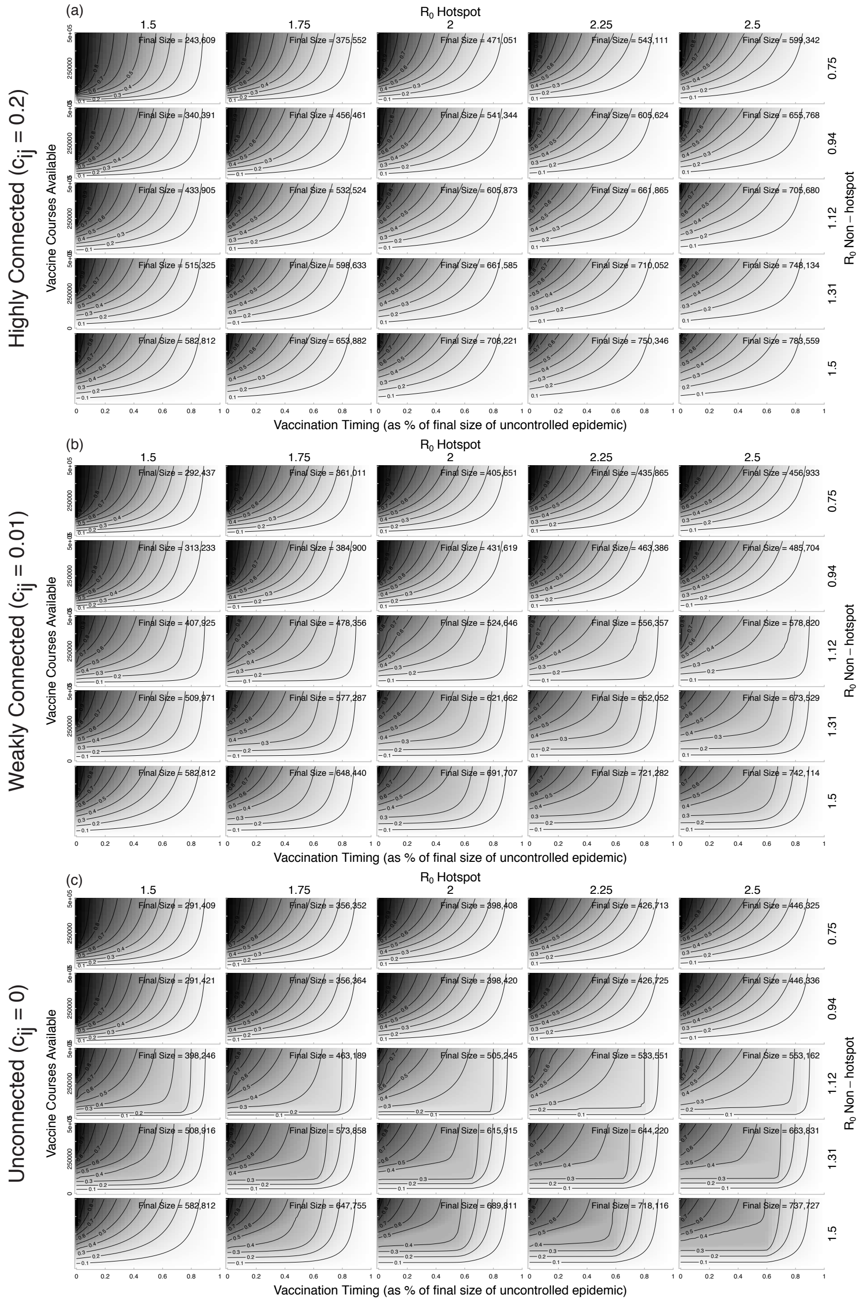

Figure S11: Percent reduction in final size compared to an uncontrolled epidemic for two-patch simulations in main analyses. Panel (a) illustrates the highly connected setting, (b) weakly connected, and (c) unconnected. Dark shading indicates larger percent reduction in final size by the best of the three vaccination strategies. Final size of the uncontrolled epidemic is shown in the top right corner of each subplot.

7 Optimal Allocations in Two Patch Models

While we only considered simple vaccination allocation strategies in the main text, here we show the optimal allocation in 2 patch systems. In all cases, the aim is to minimise the final epidemic size subject to limited vaccine supply. More formally, we wish to:

$$\text{minimise} \;\; FS = \int_{u=0}^{T} \sum_i I_i(u) du$$

$$\text{subject to} \;\; \int_{t=0}^{T} \sum_i V_i(u) du \leq V_{max}$$

where l represents area l, $V_i(u)$ represents the number vaccinated at time u in area l, and V_{max} represents the maximum number of full vaccine courses available. In the case of a two patch system this reduces to a one-dimensional constrained optimisation problem, and we used the function *minimize scalar* in the SciPy library for python [6].

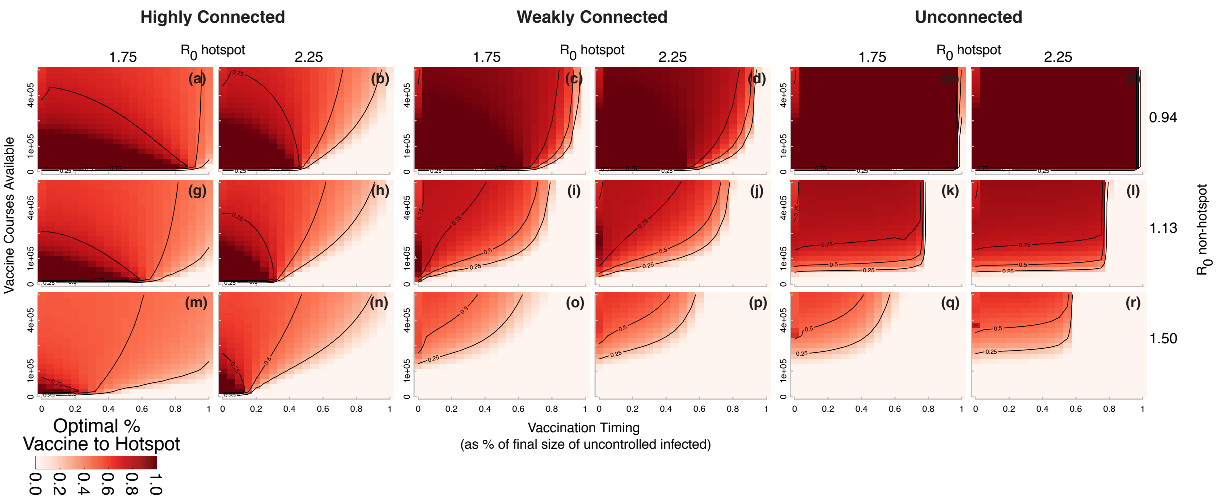

Figure S12: Optimal proportion of vaccine allocated to hotspot. Panels illustrate optimal proportion of vaccine allocated to the hotspot as a function of (1) availability of vaccine (y-axis, as a % of the total population), (2) the speed of the vaccination campaign (x-axis, as the percent of total cases infected in an uncontrolled epidemic), and (3) the transmission efficiency (as measured by the (local) reproductive number, $\mathcal{R}_i$) in each patch.

8 Results with Imperfect Vaccines

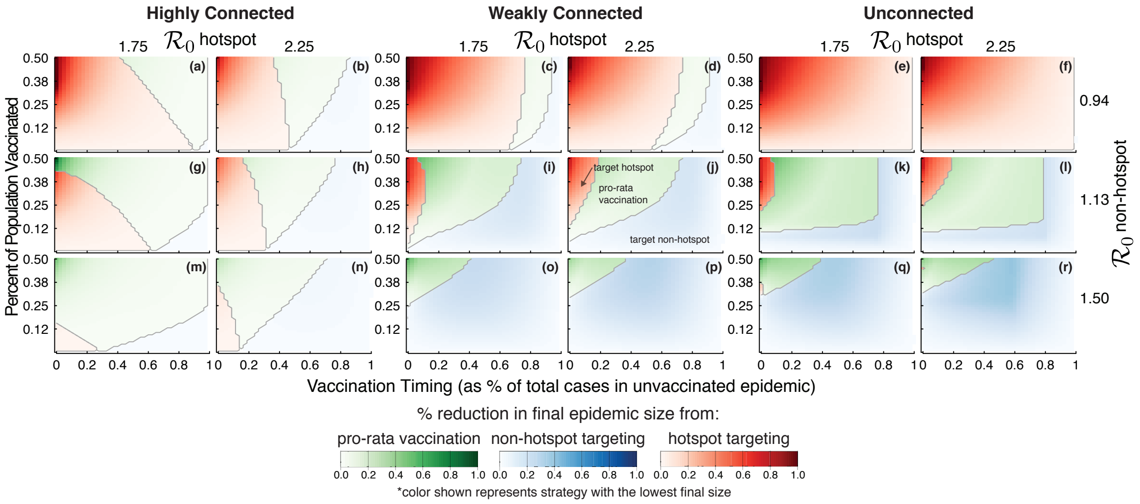

Figure S13: Overview of relative performance of vaccination strategies in 2-patch system with an imperfect vaccine (VE=60%).

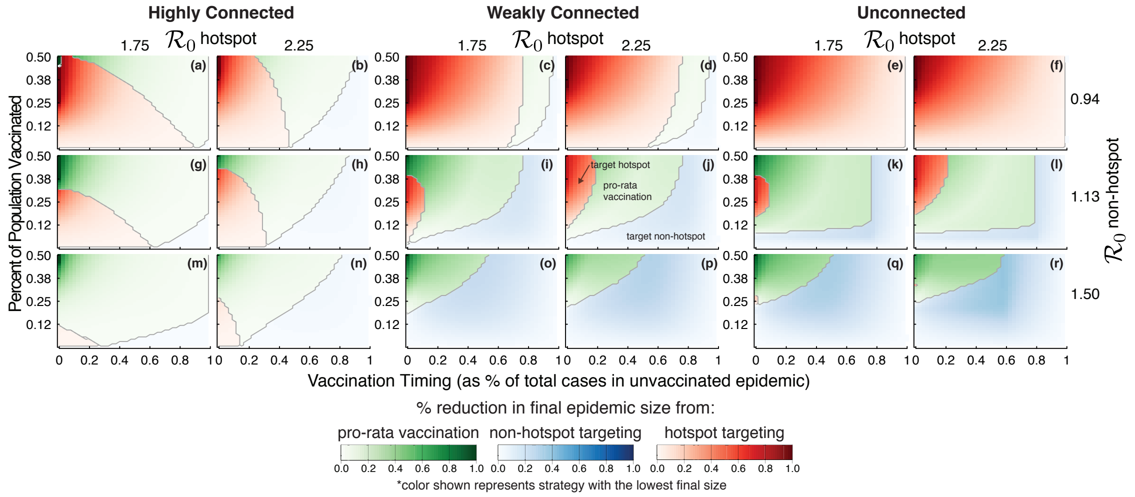

Figure S14: Overview of relative performance of vaccination strategies in 2-patch system with an imperfect vaccine (VE=80%).

References

[1] Diekmann O, Heesterbeek JAP, Roberts MG. The construction of next-generation matrices for compartmental epidemic models. Journal of The Royal Society Interface. 2010 Jun;7(47):873–885.

[2] Longini IM, Ackerman E, Elveback LR. An optimization model for influenza A epidemics. Mathematical biosciences. 1978 Jan;38(1-2):141–157.

[3] Keeling MJ, Shattock A. Optimal but unequitable prophylactic distribution of vaccine. Epidemics. 2012 Jun;4(2):78–85.

[4] Halloran ME, Longini IM, Struchiner CJ. Design and Analysis of Vaccine Studies. Springer Verlag; 2009.

[5] Wu JT, Riley S, Fraser C, Leung GM. Spatial considerations for the allocation of pre-pandemic influenza vaccination in the United States. Proceedings of the Royal Society B: Biological Sciences. 2007 Nov;274(1627):2811–2817.

[6] Oliphant TE. Python for Scientific Computing. Computing in Science & Engineering. 2007;.
